# Deuterium-Reinforced Polyunsaturated Fatty Acids Prevent Diet-Induced Nonalcoholic Steatohepatitis by Reducing Oxidative Stress

**DOI:** 10.3390/medicina58060790

**Published:** 2022-06-12

**Authors:** Haoran Li, Ouyang Zhang, Chenmin Hui, Yaxin Huang, Hengrong Shao, Menghui Song, Lingjia Gao, Shengnan Jin, Chunming Ding, Liang Xu

**Affiliations:** 1 School of Laboratory Medicine and Life Sciences, Wenzhou Medical University, Wenzhou 325035, China; tracy_731@163.com (H.L.); zoy9611@163.com (O.Z.); 13843208392@163.com (C.H.); young0504z@163.com (Y.H.); 15210972543@163.com (H.S.); menghuisong2022@163.com (M.S.); 15858831320@163.com (L.G.); 2 Key Laboratory of Laboratory Medicine, Ministry of Education, Wenzhou Medical University, Wenzhou 325035, China

**Keywords:** D-PUFA, NASH, oxidative stress, inflammation

## Abstract

*Background and Objectives:* Oxidative stress is implicated in the progression of nonalcoholic steatohepatitis (NASH) through the triggering of inflammation. Deuterium-reinforced polyunsaturated fatty acids (D-PUFAs) are more resistant to the reactive oxygen species (ROS)−initiated chain reaction of lipid peroxidation than regular hydrogenated (H−) PUFAs. Here, we aimed to investigate the impacts of D-PUFAs on oxidative stress and its protective effect on NASH. *Materials and Methods:* C57BL/6 mice were randomly divided into three groups and were fed a normal chow diet, a methionine–choline-deficient (MCD) diet, and an MCD with 0.6% D-PUFAs for 5 weeks. The phenotypes of NASH in mice were determined. The levels of oxidative stress were examined both in vivo and in vitro. *Results:* The treatment with D-PUFAs attenuated the ROS production and enhanced the cell viability in tert-butyl hydroperoxide (TBHP)−loaded hepatocytes. Concurrently, D-PUFAs decreased the TBHP-induced oxidative stress in Raw 264.7 macrophages. Accordingly, D-PUFAs increased the cell viability and attenuated the lipopolysaccharide-stimulated proinflammatory cytokine expression of macrophages. In vivo, the administration of D-PUFAs reduced the phenotypes of NASH in MCD-fed mice. Specifically, D-PUFAs decreased the liver transaminase activity and attenuated the steatosis, inflammation, and fibrosis in the livers of NASH mice. *Conclusion:* D-PUFAs may be potential therapeutic agents to prevent NASH by broadly reducing oxidative stress.

## 1. Introduction

Nonalcoholic fatty liver disease (NAFLD) is one of the most common liver diseases in the world, and it is estimated to affect approximately 25% of the global population [1]. NAFLD ranges from simple hepatic steatosis or nonalcoholic fatty liver disease (NAFL) to nonalcoholic steatohepatitis (NASH), as well as to liver fibrosis and cirrhosis, and finally to hepatocellular carcinoma [2,3]. As the global prevalence of obesity has increased in recent years, the related metabolic diseases, including NASH, are expected to continue to rise [4]. To date, in addition to lifestyle modifications, numerous medications have been added to the pipeline of novel therapies, increasing the promise of the successful treatment of NASH in the future [5]. Unfortunately, there are currently no US Food and Drug Administration (FDA)−approved therapies for NASH. Therefore, an effective therapy to treat NASH has been eagerly awaited.

Intracellular reactive oxygen species (ROS) are generated by cellular metabolic activities, such as cell survival, stress response, and inflammation [6]. In the liver, the imbalance between ROS and antioxidant molecules produces oxidative stress, which induces hepatocyte injury by inhibiting the inactivation of mitochondrial respiratory chain enzymes, glyceraldehyde-3-phosphate dehydrogenase, and membrane sodium channels [7]. In addition, ROS leads to lipid peroxidation and the production of various cytokines, which, in turn, lead to hepatocyte damage and fibrosis, and promote the progression from simple steatosis to NASH [8]. Furthermore, ROS induces the activation of resident hepatic stellate cells (HSCs), leading to liver fibrosis [9].

**Figure 1:**
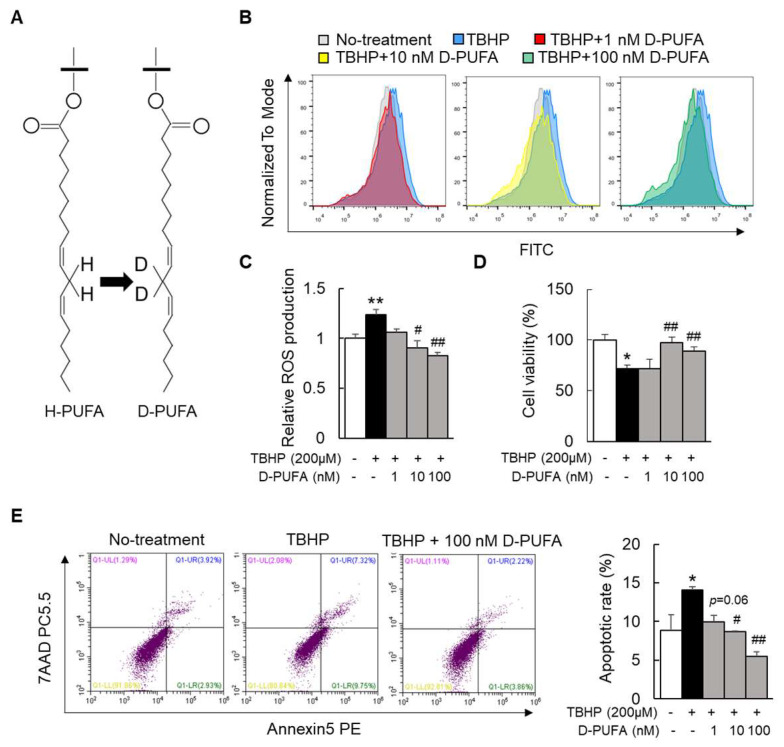
D-PUFAs attenuated oxidative stress and apoptosis induced by TBHP in hepatocytes. (**A**) Structural formula shift from H-PUFA to D-PUFA. (**B**,**C**) Relative ROS production in TBHP-treated HepG2 cells. (**D**,**E**) Cell viability and apoptotic rate in TBHP-treated HepG2 cells. Data are presented as means ± SEM, *n* = 5–6. * *p* < 0.05, ** *p* < 0.01 vs. no treatment cells; # *p* < 0.05, ## *p* < 0.01 vs. TBHP-treated cells. Significance was determined by one-way ANOVA. D-PUFA: deuterium-reinforced polyunsaturated fatty acids; H-PUFA: hydrogenated polyunsaturated fatty acids; TBHP: tert-butyl hydroperoxide; ROS: reactive oxygen species.

The nonenzymatic lipid peroxidation (LPO) of polyunsaturated fatty acids (PUFAs) induced by ROS, such as linoleic acid (LA), linolenic acid (ALA), arachidonic acid (ARA), eicosapentaenoic acid (EPA), and docosahexaenoic acid (DHA), is the main cause of oxidative damage, which leads to changes in the membrane fluidity and membrane-binding enzymes and receptors [10]. An important strategy to confine LPO is to extract bis-allyl hydrogen atoms to reduce the rate-limiting initiation step of PUFA autoxidation. This is achieved by replacing the hydrogen with deuterium at the bis-allyl position of the PUFA (from hydrogenated polyunsaturated fatty acids (H-PUFAs) to D-PUFAs (Figure 1A)) [11,12]. This substitution results in a reduced rate of PUFA peroxidation. D-PUFAs have been shown to alleviate several pathologies by restoring cellular damage, including Friedreich’s ataxia and Parkinson’s disease [13,14]. However, the effect of D-PUFAs on the development of NASH is rarely understood, and especially its role in NASH-related oxidative stress and inflammation. In the current study, we evaluated the protective effect of D-PUFAs on NASH by using a methionine–choline-deficient (MCD)−diet-induced mouse NASH model and key liver cell models.

## 2. Materials and Methods

### 2.1. Animal Model and Treatments

Eight-week-old male wild-type C57BL/6 mice (SLAC Laboratory Animal Co. Ltd., Shanghai, China), with weights of 24–25 g, were allowed to adapt to housing in the Laboratory Animal Center of Wenzhou Medical University for 1 week prior to random assignment to experimental cohorts (room temperature: 22 ± 2 °C; humidity: 45 ± 5%). After one week of acclimatization with a standard normal-chow (NC) diet and tap water ad libitum, all animals were randomly assigned to three experimental groups (*n*  =  6/group): NC, MCD diet (Dyets Inc., Bethlehem, PA, USA), and MCD diet with 0.6% D-PUFAs (Retrotope Inc., Los Altos, CA, USA). The diet ingredients are listed in Supplementary Table S1. The component of the D-PUFAs was chosen according to previous mice studies [12,15,16]. After feeding for 5 weeks, all mice were sacrificed after deep anesthesia by a peritoneal injection of pentobarbital sodium (50 mg/kg), and then the blood and the liver tissues were harvested.

The animal procedures were performed according to institutional guidelines, and this study was approved by the Wenzhou Medical University Animal Experiment Committee (wydw2019-0945), in accordance with the 3Rs policy.

### 2.2. Blood Parameters

Blood samples were collected in ethylene diamine tetra acetic acid (EDTA)−coated tubes, and plasma was stored at −80°C. Plasma levels of aspartate aminotransferase (AST) (C010-2-1), alanine aminotransferase (ALT) (C009-2-1), triglyceride (TG) (A110-1-1), and nonesterified fatty acid (NEFA) (A042-2-1) were assessed according to the instructions using colorimetric assay kits purchased from Nanjing Jiancheng Bioengineering Institute (Nanjing, China). The OD value was measured using a spectrophotometer (Varioskan Flash, Thermo Fisher Scientific, Waltham, MA, USA).

### 2.3. Liver Lipid Isolation and Determination

Liver lipids were extracted from frozen tissues via homogenization with isopropanol, as described previously [17]. The levels of TG and NEFA were examined using the above commercial kits (Jiancheng, Nanjing, China) and were normalized to the tissue weight.

### 2.4. Histopathological Analysis

The liver tissues of mice were harvested, fixed in 4% paraformaldehyde (PFA), and embedded in paraffin. The paraffin-embedded samples were then sectioned at 4 µm. The sections were stained with hematoxylin and eosin (H&E) and Sirius Red, as described previously [18]. The lipid droplets of H&E-stained liver sections and the percentage of Sirius Red positive area were quantified using ImageJ software (Version 1.8.0, National Institutes of Health, Bethesda, MD, USA).

### 2.5. Cell Culture and Treatments

HepG2 and Raw 264.7 cells were obtained from ATCC (American Type Culture Collection, Washington, DC, USA). LX2 cells were obtained from Sigma-Aldrich (Burlington, MA, USA). Cells were grown in Dulbecco’s Modified Eagle Medium (DMEM) (Gibco, Carlsbad, CA, USA), supplemented with 10% fetal bovine serum (FBS) (Gibco), 100 U/mL penicillin, and 100 μg/mL streptomycin (Pen Strep; Gibco).

HepG2 and Raw 264.7 cells were serum-starved for 6 h, co-incubated with 200 μM tert-butyl hydroperoxide (TBHP) (Sigma-Aldrich) and D-PUFA (1–100 nM) for 24 h, and harvested for cell viability, apoptosis, and ROS and qPCR analyses. Raw 264.7 and LX2 cells were serum-starved for 6 h and co-incubated with 10 ng/mL lipopolysaccharide (LPS) (Sigma-Aldrich), or 3 ng/mL transforming growth factor β (TGFβ) (R&D Systems, Minneapolis, MN, USA) and D-PUFAs (1–100 nM) for 16 h, and then harvested for qPCR analyses.

Mouse primary hepatocytes were isolated from male C57BL/6 mice (8–10 weeks old), as described previously [19]. After culturing in DMEM without FBS for 6 h, primary hepatocytes were treated with 200 μM TBHP and D-PUFAs (1–100 nM) for 24 h and were harvested for MitoSOX assay.

### 2.6. Cell Viability, Apoptosis, and ROS Assays

A Cell Counting Kit-8 (CCK-8) assay (Dojindo Laboratories, Kumamoto, Japan) was performed to measure the cell viability, following the instructions of the manufacturer. Apoptosis was evaluated using an Annexin V-PE/7-AAD Apoptosis Detection Kit (KeyGEN BioTECH, Nanjing, China) by flow cytometry. To evaluate the oxidative stress for cells, the ROS level was measured using the ROS Assay Kit (Beyotime Biotechnology, Shanghai, China) by flow cytometry. The specific steps for cell viability, apoptosis, and ROS assays were performed as described previously [20].

### 2.7. MitoSOX Assay

Superoxide radicals in mouse primary hepatocytes were estimated using the MitoSOX Red mitochondrial superoxide indicator (Invitrogen, Carlsbad, CA, USA). A total of 1.0–2.0 mL of 5 μM MitoSOX reagent working solution was applied to cover cells adhering to coverslip. Cells were incubated for 10 min at 37 °C, followed by three washes with prewarmed PBS. Cellular fluorescence intensity was detected using a confocal microscope under ambient temperature.

### 2.8. Quantitative Real-Time PCR (RT-qPCR) Analyses

Total RNA was isolated from liver tissues and cell lines using TRIzol^TM^ reagent (Invitrogen). RNA was reverse-transcribed onto cDNA using Prime-Script RT kit (Takara, Shiga, Japan), following the manufacturer’s instructions. qPCR was performed by SYBR™ Select Master Mix (Thermo Fisher Scientific). β-actin was used as the internal reference gene, and the results from RT-qPCR were analyzed through the comparative Ct method (2^−ΔΔCt^). The primer sequences are listed in Supplementary Table S2.

### 2.9. Oil Red O Staining

HepG2 cells were serum-starved for 6 h, and co-incubated with 500 μM sodium oleate (OA; Sigma-Aldrich) and D-PUFAs (1–100 nM) for 16 h. Oil Red O staining was performed to evaluate the lipid content of cells. Then, the Oil Red O dye was eluted with isopropanol, and the OD value was measured at 500 nm with a spectrophotometer (Varioskan Flash, Thermo Fisher Scientific, Waltham, MA, USA).

### 2.10. Statistical Analyses

All results are presented as mean ± standard error of the mean (SEM). A *p*-value < 0.05 was considered significant. Differences in mean values between two groups were assessed using the two-tailed Student’s *t*-test; differences in mean values among more than two groups were determined using one-way ANOVA. If results from one-way ANOVA were significant, pair-wise differences between groups were estimated using Tukey’s post hoc test. All statistical tests were performed using SPSS software (IBM Corp. Released 2019. IBM SPSS Statistics for Windows, Version 26.0. Armonk, NY, USA: IBM Corp).

## 3. Results

### 3.1. D-PUFA Attenuates Oxidative Stress and Apoptosis Induced by TBHP in Hepatocytes

To investigate the effect of D-PUFAs on oxidative stress, TBHP was used to induce HepG2 cells to establish an oxidative-stress model. Of note, the TBHP-loaded hepatocytes produced large amounts of ROS compared with the nontreatment group (Figure 1B,C). D-PUFA supplementation attenuated the TBHP-induced ROS level in a dose-dependent manner (Figure 1B,C). Moreover, the immunofluorescence staining revealed that D-PUFAs inhibited the production of ROS in the primary hepatocytes (Supplementary Figure S1). A significant reduction in the cell viability by TBHP treatment was observed, but it was reversed by the treatment with D-PUFAs (Figure 1D). Since the process of apoptosis is accompanied by the production of ROS, we next evaluated the role of D-PUFAs in apoptosis in HepG2 cells. We found that the apoptosis of HepG2 cells caused by TBHP was attenuated by treatment with D-PUFAs (Figure 1E). Therefore, these results suggest that D-PUFAs can protect cells from TBHP-mediated oxidative stress by inhibiting ROS production.

### 3.2. D-PUFAs Reduce the Oxidative Stress and Inflammation in Macrophages

**Figure 2:**
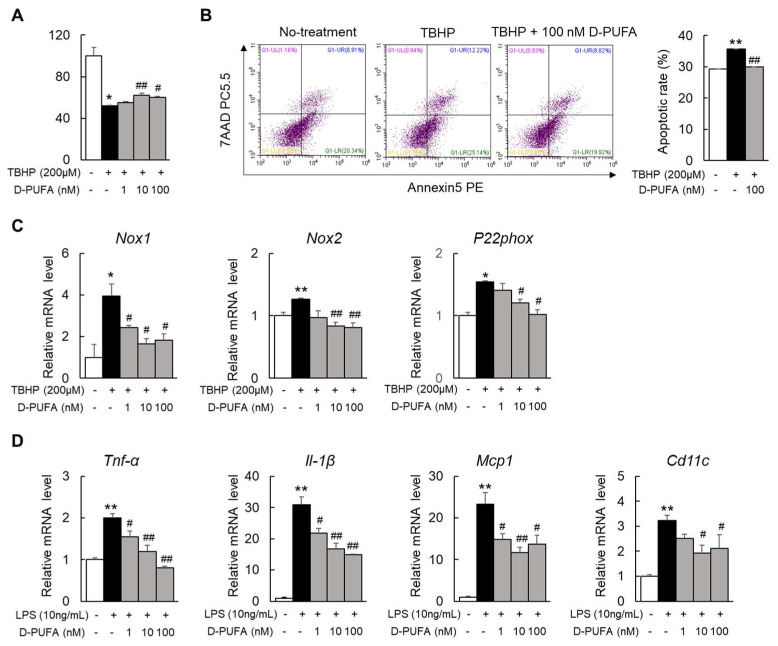
D-PUFAs reduced oxidative stress and inflammation in Raw 264.7 macrophages. (**A**,**B**) Cell viability and apoptotic rate in TBHP-treated Raw 264.7 cells. (**C**) mRNA expression of oxidative-stress-related genes in Raw 264.7 cells. (**D**) mRNA expression of LPS-induced inflammatory factors in Raw 264.7 cells. Data are presented as means ± SEM, *n* = 5–6. * *p* < 0.05, ** *p* < 0.01 vs. no treatment cells; # *p* < 0.05, ## *p* < 0.01 vs. TBHP- or LPS-stimulated condition. Significance was determined by one-way ANOVA. TBHP: tert-butyl hydroperoxide; D-PUFA: deuterium-reinforced polyunsaturated fatty acids; LPS: lipopolysaccharide; Nox1: nicotinamide adenine dinucleotide phosphate oxidase 1; Nox2: nicotinamide adenine dinucleotide phosphate oxidase 2; P22phox: NADPH oxidase subunit p22phox; Tnf-α: tumor necrosis factor α; Il-1β: interleukin 1 β; Mcp1: monocyte chemoattractant protein 1; Cd11c: integrin alpha X.

In addition to hepatocytes, oxidative stress also triggers apoptosis and inflammation in liver macrophages [21]. Thus, we next selected the Raw 264.7 cells, a mouse monocyte–macrophage cell line, as the study object. The results were consistent with those in HepG2 cells: D-PUFAs also markedly restored the decrease in the cell viability and reduced the apoptotic rate in TBHP-stimulated Raw 264.7 cells (Figure 2A,B). In addition, the mRNA levels of the NADPH oxidase (*Nox1*), *Nox2*, and the critical component of the NOX system, *P22phox*, were increased by treatment with TBHP. Unsurprisingly, the D-PUFA treatment significantly decreased the mRNA levels of these oxidases in TBHP-treated cells (Figure 2C). Extensive previous research has revealed the mechanisms by which persistent oxidative stress can lead to inflammation, which, in turn, mediates multiple chronic diseases [22]. LPS is a common endotoxin that can activate a series of inflammatory pathways in vitro to synthesize and release a variety of cytokines and inflammatory mediators [23]. Likewise, the use of D-PUFAs significantly reduced the mRNA levels of these inflammatory factors (Figure 2D). Together, these results indicate that D-PUFAs alleviate inflammation by reducing oxidative stress in macrophages.

### 3.3. D-PUFAs Prevent MCD-Induced Steatosis in Mice

**Figure 3:**
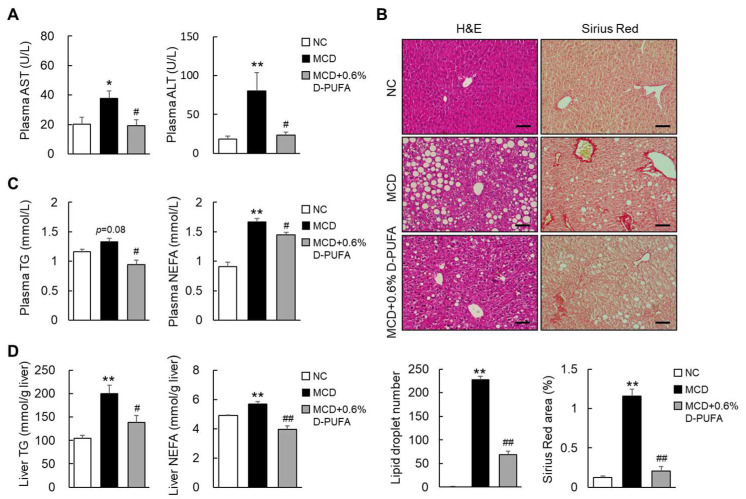
D-PUFAs prevent NASH progression in MCD mice. (**A**) Plasma levels of AST and ALT. (**B**) Representative photographs of H&E and Sirius Red staining of liver sections. Scale bars = 100 μm. (**C**) Plasma levels of TG and NEFA. (**D**) Levels of hepatic TG and NEFA contents. Data are presented as means ± SEM, *n* = 5–6. * *p* < 0.05, ** *p* < 0.01 vs. NC; # *p* < 0.05, ## *p* < 0.01 vs. MCD. Significance was determined by one-way ANOVA. AST: aspartate aminotransferase; ALT: alanine aminotransferase; NC: normal chow; MCD: methionine-choline-deficient; D-PUFA: deuterium-reinforced polyunsaturated fatty acids; H&E: hematoxylin and eosin; TG: triglyceride; NEFA: nonesterified fatty acid; NASH: nonalcoholic steatohepatitis.

To investigate the hepatoprotective effect of D-PUFAs in NASH, we treated the MCD-fed mice with 0.6% D-PUFAs for five weeks. The results show that the MCD diet induced liver injury, which manifested as increased levels of plasma transaminases, such as ALT and AST. As expected, the D-PUFA treatment significantly reduced the ALT and AST levels in the MCD-diet-fed mice (Figure 3A). Concurrently, the D-PUFAs significantly attenuated the MCD-diet-induced hepatic steatosis, which manifested as a reduction in ballooning and smaller lipid vacuoles (Figure 3B). Consistently, D-PUFAs markedly reduced the levels of TG and NEFA in both the plasma and livers of MCD-fed mice (Figure 3C,D). In vitro, Oil Red O staining showed that D-PUFAs also attenuated lipid accumulation in HepG2 cells (Supplementary Figure S2). Moreover, the Sirius Red staining revealed that D-PUFAs also reduced the MCD-diet-induced liver fibrosis in mice (Figure 3B). These results suggest that D-PUFAs mitigate the phenotypes of NASH in mice.

### 3.4. D-PUFAs Attenuate Oxidative Stress and Inflammation Induced by MCD Diet in Mice

**Figure 4:**
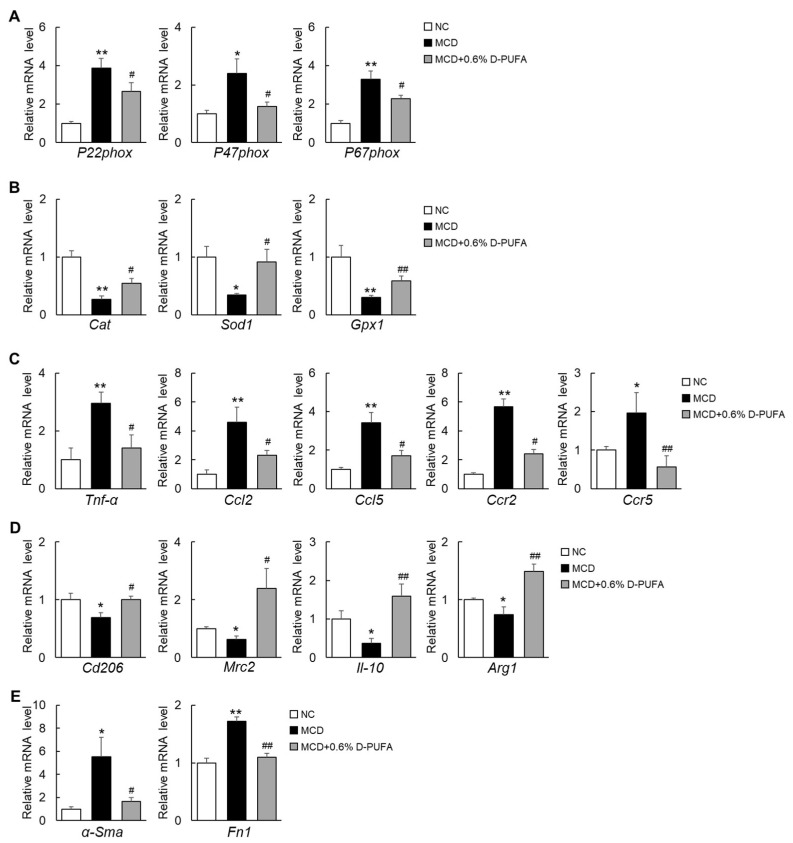
D-PUFAs decrease oxidative stress and inflammation in the liver of NASH mice. (**A**) mRNA expression of oxidative-stress-related genes in the liver. (**B**) mRNA expression of antioxidative-stress-related genes in the liver. (**C**) mRNA expression of inflammation genes in the liver. (**D**) mRNA expression of anti-inflammation genes in the liver. (**E**) mRNA expression of fibrosis genes in the liver. Data are presented as means ± SEM, *n* = 5–6. * *p* < 0.05, ** *p* < 0.01 NC; # *p* < 0.05, ## *p* < 0.01 vs. MCD. Significance was determined by one-way ANOVA. NC: normal chow; MCD: methionine–choline-deficient; D-PUFA: deuterium-reinforced polyunsaturated fatty acids; P22phox: NADPH oxidase subunit p22phox; P47phox: NADPH oxidase subunit p47phox; P67phox: NADPH oxidase subunit p67phox; Cat: catalase; Sod1: superoxide dismutase 1; Gpx1: glutathione peroxidase 1; Tnf-α: tumor necrosis factor α; Ccl2: C-C motif chemokine ligand 2; Ccl5: C-C motif chemokine ligand 5; Ccr2: C-C motif chemokine receptor 2; Ccr5: C-C motif chemokine receptor 5; Cd206: mannose receptor C type 1; Mrc2: mannose receptor C type 2; Il-10: interleukin 10; Arg1: arginase 1; α-Sma: α-smooth muscle actin; Fn1: fibronectin 1.

The “Multi-hit-hypothesis” revealed that oxidative-stress-mediated inflammation is involved in the pathogenesis of NASH [24]. In the current study, we found that the production of peroxidase, which is represented by the NADPH oxidase subunit, was significantly increased in MCD-diet-fed mice, whereas these subunits were significantly reduced by D-PUFAs (Figure 4A). In contrast, the expression of antioxidative-stress-associated genes was significantly elevated during the D-PUFA treatment (Figure 4B). Moreover, the expressions of proinflammatory cytokines and chemokines, including *Tnf-α*, *Ccl2*, *Ccl5*, *Ccr2,* and *Ccr5*, were markedly decreased by D-PUFAs (Figure 4C). In contrast, the expressions of anti-inflammatory markers (*Cd206*, *Mrc2*, *Il-10*, and *Arg1*) were upregulated in the livers of the MCD + D-PUFA mice (Figure 4D). In line with the attenuation of fibrogenesis, D-PUFAs decreased the mRNA levels of *α-Sma* (a reliable marker of hepatic stellate cells) and fibronectin 1 (*Fn1*) (Figure 4E). Accordingly, D-PUFAs also reduced the expression of *α-Sma* and collagen type I alpha 1 (*Col1α1*) in TGFβ−treated human LX2 stellate cells (Supplementary Figure S3). These results suggest that D-PUFAs prevent the development or progression of NASH, at least in part by inhibiting oxidative stress and inflammation.

## 4. Discussion

Our study demonstrates that D-PUFAs attenuated the NASH-related steatosis, inflammation, and fibrosis in MCD-induced mice. Mechanistically, the D-PUFAs may have acted on both the parenchymal and nonparenchymal cells of the liver in the NASH mice model. Specifically, the D-PUFAs inhibited the fat deposition in hepatocytes, reduced the oxidative stress and inflammation in macrophages, and finally suppressed the activation of stellate cells, thereby preventing the progression of NASH.

Recently, many studies have shown the importance of oxidative stress in the progression of simple fatty liver to steatohepatitis [25,26,27]. The continuous generation of ROS in NASH mainly comes from abnormal mitochondria in liver cells, the abnormal synthesis and secretion of several antioxidant enzymes, and the depletion of glutathione [28]. The overproduction of ROS inhibits the ability of other antioxidant defense systems in NASH and leads to further oxidative damage. Clinical studies have confirmed that the level of ROS in NASH patients is elevated and correlated with the degree of liver damage [29,30,31]. Vitamin E, as a natural scavenging reactive oxygen species, is currently believed to be effective against NASH in a successful pilot study [32]. In addition, adropin protein also protects against liver damage in NASH through its antioxidant capacity [33].

In recent years, a variety of PUFAs, including n-3 and n-6 H-PUFA, have been well documented to have beneficial effects on NASH [34,35,36]. However, the n-3 H-PUFA (from diet or converted from n-6 H-PUFA) has been shown to have deleterious effect on NASH because it is sensitive to lipid peroxidation and generates oxidative stress in the liver [37]. D-PUFAs are more resistant to the ROS-initiated chain reaction of lipid peroxidation than regular H-PUFAs [11]. Indeed, compared with H-PUFAs, D-PUFAs have shown better therapeutic effects in various diseases [38,39]. D-PUFAs can prevent atherosclerosis by reducing LPO and hypercholesterolemia [38]. Additionally, D-PUFAs improved the cognitive performance in models of Alzheimer’s and Huntington’s diseases by reducing LPO-mediated oxidative stress [12,15]. In the current study, the D-PUFA treatment reduced the upregulation of the mRNA levels of the NADPH oxidase subunits in the liver of a NASH mouse model. Moreover, the use of D-PUFAs also reduced the expression of the NADPH oxidase mRNA level in a cellular model of TBHP-induced oxidative stress. Therefore, the improvement in NASH by D-PUFAs may be mainly achieved by inhibiting oxidative stress.

In the development of NASH, oxidative stress activates multiple transcription factors, resulting in the abnormal expression of certain genes in inflammatory pathways that contribute to many chronic diseases, including NASH [40,41]. The activation of macrophages by inflammatory cytokines or LPS is a major process in inflammation-related diseases [42]. Specifically, LPS activates proinflammatory signals through its pattern-recognition receptor, toll-like receptor 4 (TLR4), to promote NASH and its associated fibrosis [24,43]. Macrophages, in turn, enhance the nuclear translocation of inflammatory cytokines, nuclear factor kappa B (NF-κB), and the activation of mitogen-activated protein kinase (MAPK) [44]. During the development of NASH, liver injury triggers the activation of Kupffer cells, which are liver-resident macrophages, which leads to the release of inflammatory cytokines and chemokines [45]. In the current study, the data show that D-PUFAs attenuated the expression of proinflammatory factors in the livers of MCD-diet-fed mice and in LPS-stimulated Raw 264.7 cells. Therefore, D-PUFAs not only inhibit oxidative stress, but also improve the resulting inflammation, thereby preventing the development of NASH.

Liver fibrosis is a major determinant of progression to cirrhosis and mortality in NASH patients, and especially the deposition of an extracellular matrix (ECM) that is rich in type I collagen [46]. Activated HSCs in the liver secrete ECM proteins through oxidative stress and inflammatory stimulation, leading to fibrous scarring [47]. The alleviation of liver fibrosis is an important part of the treatment of NASH. Our results show that D-PUFAs attenuated the collagen deposition and mRNA expression of profibrotic growth factors (*α-Sma* and *Col1α1*) in the liver of MCD-diet-fed mice, suggesting that D-PUFAs protect the liver from fibrosis by inhibiting HSC activation.

## 5. Conclusions

There are several shortcomings in this study that need to be examined in more detail in the future. Although the MCD-induced NASH model is classic, it is toxic and causes weight loss in mice. Therefore, other NASH mice models, such as high-fat diet-induced NASH mice, should be used to further confirm the beneficial effects of D-PUFAs on NASH. In addition, whether the protective effect of D-PUFAs on NASH is dose-dependent also needs to be addressed. Most importantly, the advantages of D-PUFAs in preventing NASH progression compared with H-PUFAs are for further study. Nevertheless, the present study demonstrates that D-PUFAs could prevent steatosis and liver damage in NASH mice by inhibiting hepatic oxidative stress, which suggests that D-PUFAs may be a potential approach to the treatment of NASH.

## Data Availability

The authors confirm that the data supporting the findings of this study are available within the article and/or its Supplementary Materials.

## Supplementary Materials

The following supporting information can be downloaded at: https://www.mdpi.com/article/10.3390/medicina58060790/s1, Supplementary Figure S1: D-PUFA decreases the MitoSOX in primary hepatocytes; Supplementary Figure S2: D-PUFA reduces the lipid accumulation in hepatocytes; Supplementary Figure S3: D-PUFA attenuates HSC activity in LX2 stellate cells; Supplementary Table S1: The diets ingredient; Supplementary Table S2: Primers used for RT-qPCR.
