# Supplementary material for: Deuterium-Reinforced Polyunsaturated Fatty Acids Prevent Diet-Induced Nonalcoholic Steatohepatitis by Reducing Oxidative Stress
Source: Medicina (Kaunas). 2022 Jun 12;58(6):790. doi: 10.3390/medicina58060790 (PMC9228393; doi:10.3390/medicina58060790)
Supplement: Supplementary file 1 [file medicina-58-00790-s001.zip › medicina-1747373-supplementary.pdf]

Supplementary Tables:

Supplementary Table S1: The diets ingredient.

Product #	519580		New Diet	
**Ingredient**	**grams/kg**	**Kcal/kg**	**grams/kg**	**Kcal/kg**
L-Alanine	3.5	14	3.5	14
L-Arginine	12.1	48	12.1	48
L-Asparagine-H_2O	6	24	6	24
L-Aspartate	3.5	14	3.5	14
L-Cystine	3.5	14	3.5	14
L-Glutamine	40	160	40	160
Glycine	23.3	93	23.3	93
L-Histidine-HCI-H20	4.5	18	4.5	18
L-Isoleucine	8.2	33	8.2	33
L-Leucine	11.1	44	11.1	44
L-Lysine-HCI	18	72	18	72
L-Phenylalanine	7.5	30	7.5	30
L-Proline	3.5	14	3.5	14
L-Serine	3.5	14	3.5	14
L-Threonine	8.2	33	8.2	33
L-Tryptophan	1.8	7	1.8	7
L-Tyrosine	5	20	5	20
L-Valine	8.2	33	8.2	33
Total amino acids	171.4	686	171.4	686
Sucrose	455.3	1821.2	455.3	1821.2
Cornstarch	150	600	150	600
Dyetrose	50	200	50	200
Corn Oil	150	600	150	600
Cellulose	30	0	30	0
Mineral Mix #200000	35	0	35	0
Sodium Bicarbonate	7.5	0	7.5	0
Vitamin Mix #300050	10	40	10	40
Choline Bitartrate	0	0	0	0
L-Methionine	0	0	0	0
D-PUFA	0	0	6.056	0
Red Dye	0.05	0	0.05	0
Total	1009.25	4247	1015.306	4247

Supplementary Table S2: Primers used for RT–qPCR.

Primer	Organism	5'-Sequence-3'
β-actin-F	mouse	AGGCCCAGAGCAAGAGAGGTA
β-actin-R	mouse	GGGGTGTTGAAGGTCTCAAACA
Nox1-F	mouse	GGAATTGCAGATGAGGAAGC
Nox1-R	mouse	CCCAACCAGTACAGCCACTT
Nox2-F	mouse	TTGGGTCAGCACTGGCTCTG
Nox2-R	mouse	TGGCGGTGTGCAGTGCTATC
P22phox-F	mouse	GTCCACCATGGAGCGATGTG
P22phox-R	mouse	CAATGGCCAAGCAGACGGTC
P47phox-F	mouse	GATGTTCCCCATTGAGGCCG
P47phox-R	mouse	GTTTCAGGTCATCAGGCCGC
P67phox-F	mouse	CTGGCTGAGGCCATCAGACT
P67phox-R	mouse	AGGCCACTGCAGAGTGCTTG
Cat-F	mouse	CCAGCGACCAGATGAAGCAG
Cat-R	mouse	CCACTCTCTCAGGAATCCGC
Sod1-F	mouse	CAGCATGGGTTCCACGTCCA
Sod1-R	mouse	CACATTGGCCACACCGTCCT
Gpx1-F	mouse	TTCGGACACCAGGAGAATGG
Gpx1-R	mouse	TAAAGAGCGGGTGAGCCTTC
Tnf-α-F	mouse	AAGCCTGTAGCCCACGTCGTA
Tnf-α-R	mouse	GGCACCACTAGTTGGTTGTCTTTG
Il-1β-F	mouse	CTGAACTCAACTGTGAAATGCCA
Il-1β-R	mouse	AAAGGTTTGGAAGCAGCCCT
Mcp1-F	mouse	AGGTCCCTGTCATGCTTCTGG
Mcp1-R	mouse	CTGCTGCTGGTGATCCTCTTG
Cd11c-F	mouse	AAAATCTCCAACCCATGCTG
Cd11c-R	mouse	CACCACCAGGGTCTTCAAGT
Ccl5-F	mouse	TGCCCTCACCATCATCCTCACT
Ccl5-R	mouse	GGCGGTTCCTTCGAGTGACA
Ccr2-F	mouse	ATTCTCCACACCCTG TTT
Ccr2-R	mouse	GATTCCTGGAAGGTG
Ccr5-F	mouse	ATTCTCCACACCCTGTTT
Ccr5-R	mouse	CGTTTGACCATGTGT
Cd206-F	mouse	CAAGGAAGGTTGGCATTTGT
Cd206-R	mouse	CCTTTCAGTCCTTTGCAAGC
Mrc2-F	mouse	TACAGCTCCACGCTATGG
Mrc2-R	mouse	CACTCTCCCAGTTGA
Il-10-F	mouse	GCTCTTACTGACTGGCATGAG
Il-10-R	mouse	CGCAGCTCTAGGAGCATGTG
Arg1-F	mouse	CTCCAAGCCAAAGTCCTTAGAG
Arg1-R	mouse	AGGAGCTGTCATTAGGGACATC
α-Sma-F	mouse	TGTGCTGGACTCTGGAGA
α-Sma-R	mouse	GAAGGAATAGCCACG

Fn1-F	mouse	TGCACGATGATATGGAGAGC
Fn1-R	mouse	TGGGTGTCACCTGACTGAAC
β-actin-F	human	CACCATTGGCAATGAGCGGTTC
β-actin-R	human	AGGTCTTTGCGGATGTCCACGT
α-Sma-F	human	TCATGGTCGGTATGGGTCAG
α-Sma-R	human	CGTTGTAGAAGGTGTGGTGC
Col1α1-F	human	GAGGGCAACAGCAGGTTCACTTA
Col1α1-R	human	TCAGCACCACCGATGTCCA

Supplementary Figures

Supplementary Figure S1

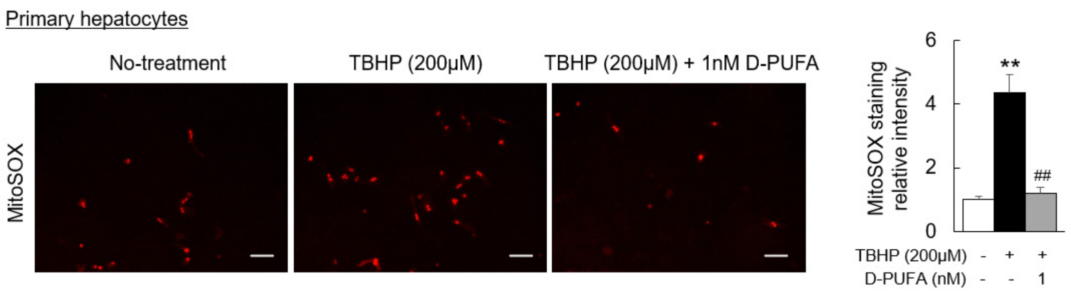

Supplementary Figure S1. D−PUFA decreases the MitoSOX in primary hepatocytes. Relative MitoSOX in primary hepatocytes. Scale bars = 100 μm. Data are presented as means ± SEM, n = 5−6. ** $p < 0.01$ vs. no treatment cells, ## $p < 0.01$ vs. TBHP−treated cells. Significance was determined by one-way ANOVA. TBHP, tert−butyl hydroperoxide; D−PUFA, deuterium−reinforced polyunsaturated fatty acids.

Supplementary Figure S2

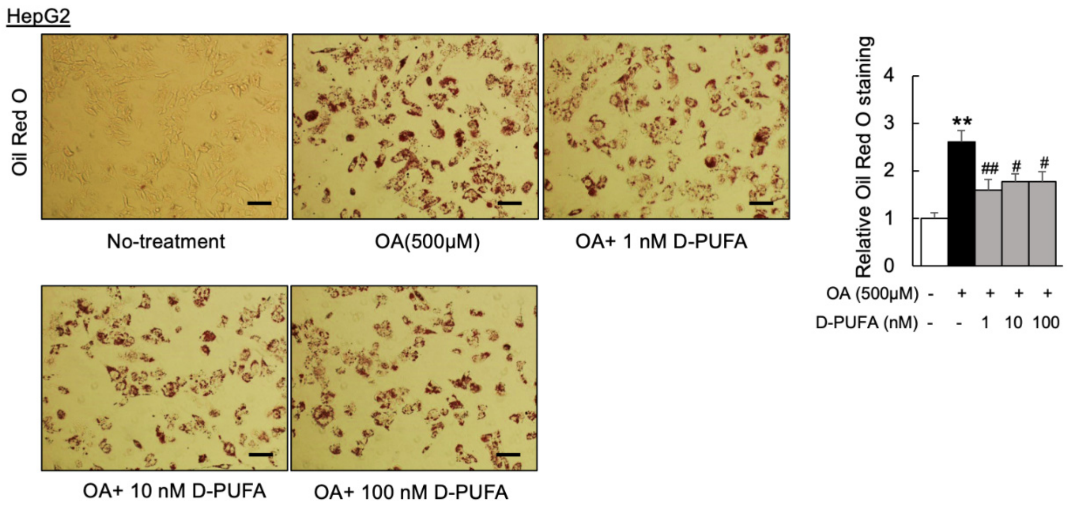

Supplementary Figure S2. D−PUFA reduces the lipid accumulation in hepatocytes. Oil Red O staining of HepG2 cells. Scale bars = 100 μm. Data are presented as means ± SEM, $n = 5–6$. ** $p < 0.01$ vs. no treatment cells, $^{\#}p < 0.05$, $^{\#\#}p < 0.01$ vs. OA−stimulated condition. Significance was determined by one-way ANOVA. OA, sodium oleate; D−PUFA, deuterium−reinforced polyunsaturated fatty acids.

Supplementary Figure S3

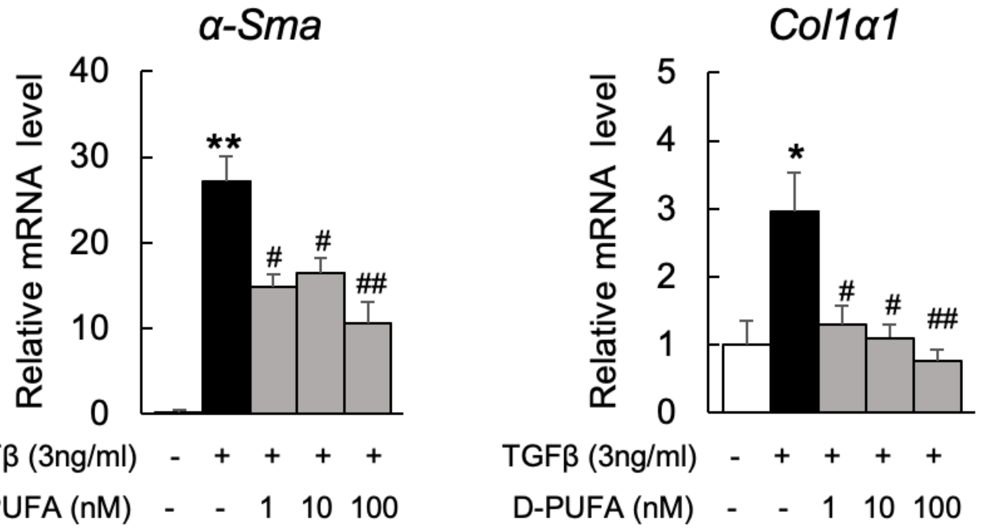

Supplementary Figure S3. D−PUFA attenuates HSC activity in LX2 stellate cells. mRNA expression of fibrosis genes in LX2 cells. Data are presented as means ± SEM, $n = 4–6$. * $p < 0.05$, ** $p < 0.01$ vs. no treatment cells, $^{\#}p < 0.05$, $^{\#\#}p < 0.01$ vs. TGFβ−stimulated condition. Significance was determined by one-way ANOVA. TGFβ, transforming growth factor β; D−PUFA, deuterium−reinforced polyunsaturated fatty acids; α-Sma, α-smooth muscle actin; Col1α1, collagen type I α 1.
